# Cochlear implantation in deafblind patients: a systematic review and meta-analysis

**DOI:** 10.1007/s00405-026-10280-2

**Published:** 2026-07-19

**Authors:** Ahmed Sobhy, Tawfiq Khurayzi, Ahmed Nazmy, Hussein A. El-Shirbeny, Mahmoud Abd El-Nasser, Abdullah Sobhy, Eslam Radwan, Reem F. Suliman, Mo’tasem Abdelaziz, Mennatullah Ashour, Nehal Ataky, Saad Elzayat, Edoardo Covelli, Alsafa Khalil, Haitham H. Elfarargy

**Affiliations:** 1 Faculty of Medicine, Kafr El-Shiekh University, Kafr El-Shiekh, Egypt; 2 Cochlear Implant Center, Jazan Health Cluster, Jazan, Saudi Arabia; 3 Otorhinolaryngology Department, Faculty of Medicine, Kafr El-Shiekh University, Kafr El-Shiekh, Egypt; 4 College of Human Medicine, Banha University, Banha, Egypt; 5 Cardiology Department, El Arish General Hospital, North Sinai, El Arish, Egypt; 6 Department of Neuroscience, Mental Health and Sensory Organs, Faculty of Medicine and Psychology, Sant’Andrea University Hospital, Sapienza University, Rome, Italy; 7 Alexandria School of Medicine, Alexandria, Egypt

**Keywords:** Cochlear implantation, Deafblindness, Usher syndrome, Speech intelligibility, Auditory rehabilitation, Dual sensory loss

## Abstract

**Purpose:**

In the past, cochlear implantation (CI) was not commonly offered to this population due to concerns about limited auditory rehabilitation outcomes. Recent advancements in CI technology and multidisciplinary rehabilitation have expanded candidacy, but outcomes in deafblind patients remain underexplored. This systematic review and meta-analysis aimed to synthesize available evidence on CI outcomes in deafblind individuals.

**Methods:**

Data sources included PubMed, Web of Science, Scopus, and Cochrane Library from inception to July 20, 2025. Observational studies included adults or children with confirmed dual sensory impairment after CI. Primary outcomes included speech clarity (Speech Intelligibility Rating [SIR]), verbal communication ability, and telephone communication competence. Secondary outcomes encompassed audiological performance, auditory and cognitive development, educational integration, psychological benefits, and advanced auditory skills. Risk of bias was examined using the NIH Quality Assessment Tool and Newcastle–Ottawa Scale. STATA 18 was used to perform random-effects meta-analyses.

**Results:**

Thirty-six studies were analyzed, including 491 patients (mean age 23.68 years; 23% male). The pooled mean SIR score was 3.45 (95% CI: 2.69–4.21), with no significant difference between Usher and non-Usher groups (*p* = 0. 52). Verbal communication ability was achieved in 70% (95% CI: 0.60–0.79), while telephone communication competence was limited to 51% (95% CI: 0.33–0.69).

**Conclusion:**

Cochlear implantation is an effective intervention for deafblind individuals, demonstrating significant improvements in speech intelligibility and verbal communication regardless of the underlying etiology (Usher vs. non-Usher). While verbal communication rates are promising, advanced skills such as telephone competence remain limited, suggesting the need for continued multidisciplinary rehabilitation.

## Introduction

Deafness in early childhood is a significant health concern, with one in every 1000 children born deaf. Hearing loss can be an isolated condition or part of a syndrome in about 30% of cases. Syndromes such as Usher, CHARGE, and Alström frequently involve progressive visual dysfunction, compounding communication and developmental challenges. In contrast, Waardenburg syndrome is typically associated with pigmentary abnormalities and sensorineural hearing loss and is *not* characteristically linked to reduced visual acuity [1].

The frequency of hearing loss and blindness together varies. Usher syndrome is the most common genetic cause of combined hearing and vision loss, accounting for nearly half of individuals with deafblindness. It is an autosomal recessive condition characterized by congenital or early-onset sensorineural hearing loss and progressive retinitis pigmentosa, with clinical subtypes distinguished by auditory severity and vestibular involvement [2, 3]. Other syndromes, like Alstrom syndrome, are much rarer, with an incidence of 1 in 1,000,000 births [4].

Individuals with sensorineural hearing loss who derive insufficient benefit from conventional hearing aids may benefit from cochlear implantation to rehabilitate auditory perception. A cochlear implant sends electrical signals directly to the auditory nerve, bypassing the damaged parts of the inner ear. The benefits of CIs on communication, language development, and quality of life are well-established. In the past, children with additional disabilities were not considered candidates for cochlear implants [5]. Recently, however, the criteria for pediatric cochlear implantation have become more flexible, allowing some children with complex needs to be considered. The main reason for this change is the increased experience of rehabilitation centers, which now expect better outcomes in these children. Deaf children with other disabilities can see a lot of benefits from a cochlear implant, not just in their hearing but also in communication and language skills. How well they do depend on things like age at the time of implantation, how long they have been deaf, and the type of rehabilitation they received before the surgery [6–8].

Despite the growing body of research on CI outcomes in various patient groups, no systematic review has explicitly focused on cochlear implantation in deafblind patients. This gap in the literature makes it difficult to get a complete picture of the results of CI for this population. Therefore, this is the first systematic review to examine the existing literature on cochlear implantation in deafblind patients, including those with syndromes like Usher, Alstrom, and CHARGE, to better understand this intervention’s outcomes.

## Methods

### Protocol and registration

This systematic review and meta-analysis were conducted in accordance with the Preferred Reporting Items for Systematic Reviews and Meta-Analyses (PRISMA) guidelines [9] and adhered to the rigorous methodological standards outlined in the Cochrane Handbook for Systematic Reviews of Interventions [10]. The study protocol was prospectively registered in the International Prospective Register of Systematic Reviews (PROSPERO) under the registration number (CRD420251110489).

### Data sources & search strategy

To identify relevant studies for this review, we conducted a comprehensive systematic search across major electronic databases, including PubMed (MEDLINE), Web of Science (WOS), SCOPUS, and Cochrane Library from inception until July 20th, 2025, using the following search terms: [“Cochlear Implantation” OR “Cochlear Implants” OR “Auditory Prosthesis”] AND [“Deaf blindness” OR “Deaf-Blind” OR “Dual Sensory Loss” OR “Usher syndrome”]. Full search strategies tailored for each database are provided in the **Supplemental Table 1**. To ensure that the literature search was comprehensive, backward and forward citation analysis using snowball methodology was performed on the included studies and relevant meta-analyses to ensure inclusion of all relevant studies.

### Screening and selection process

Following the removal of duplicates using EndNote, the remaining articles were imported into the Rayyan platform for systematic evaluation [11]. The selection process involved two sequential phases. Initially, two independent reviewers screened the titles and abstracts to identify studies that met the preliminary eligibility criteria. Subsequently, the full texts of potentially relevant articles were assessed in detail to confirm their eligibility for inclusion. Any discrepancies between reviewers were addressed through discussion, and when agreement could not be reached, a third reviewer was consulted to resolve the disagreement and maintain impartiality in study selection.

### Eligibility criteria and outcomes

We included only observational studies (e.g., case series, cohort, or case-control) involving adults or children with a confirmed diagnosis of dual sensory impairment (deaf-blindness). Deafblindness was operationally defined as the coexistence of significant hearing loss requiring cochlear implantation and documented visual impairment affecting communication or daily functioning, as reported by the original studies. Studies including individuals with partial visual impairment were included if the visual deficit substantially limited communication abilities. Eligible studies assessed patients who underwent cochlear implantation (CI), with or without a comparison group, and reported outcomes of interest based on an intention-to-treat (ITT) analysis. On the other hand, we excluded editorials, duplicate studies, full-text unavailable, case reports, or conference abstracts.

The primary outcomes were Speech & Communication Outcomes, including Speech Clarity (Speech Intelligibility Rating [SIR] Scale), Verbal Communication Ability (mode of communication by speech), and Telephone Communication Competence (ability to communicate via telephone effectively). Secondary outcomes included: [1] Audiological Performance Outcomes: Hearing Threshold Levels (Pure Tone Audiometry [PTA, dB HL]), Speech Perception Accuracy (Word Recognition Score), and Environmental Sound Detection (Environmental Sound Recognition tests); [2] Auditory & Cognitive Development Outcomes: Auditory Processing Skills (Categories of Auditory Performance [CAP] Scale) and Cognitive Functionality (assessment of normal cognitive skills); [3] Educational & Social Adaptation: Educational Integration Success (mainstream participation in hearing aid unit schooling); [4] Quality of Life & Psychosocial Benefits: Quality of Life Improvement (Glasgow Benefit Inventory [GBI]) and Family/Social Support Impact (good family relationships); [5] Advanced Auditory & Musical Skills: Musical Perception Ability (piano playing) and Media Engagement (daily radio listening).

### Data extraction and risk of bias assessment

Four authors independently extracted data using a standardized data extraction form in Microsoft Excel. All data were cross verified for accuracy. Extracted variables included study characteristics, patient demographics, and reported outcomes.

Two independent authors assessed the risk of bias in the included studies. We used the Newcastle–Ottawa Scale (NOS) for comparative studies [12], while the NIH Quality Assessment Tool was used for single-arm studies [13]. For NOS, a maximum of nine stars was given to each study; higher ratings denoted a lesser probability of bias, while for NIH quality rating was as follows: <50% indicates poor quality, 50–75% indicates fair quality, and ≥ 75%. Indicates good quality. Discrepancies were resolved by discussion with a third author.

### Statistical analysis and heterogeneity assessment

A meta-analysis was performed using a random-effect model with statistical pooling of data based on the Der Simonian–Laird method to estimate the pooled results of dichotomous outcomes by employing the proportion estimate. In contrast, we used the post-intervention mean values for continuous outcomes, both with a 95% confidence interval (CI). In cases where studies reported long-term follow-up data at multiple time points, the last follow-up point was selected for inclusion in the analysis. The quantitative analysis was conducted using STATA MP version 18, utilizing appropriate statistical packages for computation and data visualization. The Chi-square test (specifically, the Cochran Q test) was used to assess statistical heterogeneity across the studies. The evaluation was conducted using the following equation: [**I**^**2**^= (Q − dfQ) x 100%]. A P-value of less than 0.1 for the Chi-square test was considered indicative of significant heterogeneity, and I² values ≥ 50% were interpreted as high heterogeneity. The Galbraith plot was also used to identify outlier studies [14]. To assess the possible publication bias, we performed a funnel plot to visualize the relationship between pooled effect sizes and the standard error [15].

Subgroup analyses were performed based on study design (retrospective case series, retrospective cohort, and case-control studies) and population type (Usher syndrome vs. non-Usher syndrome) to explore potential sources of heterogeneity and assess possible effect modification.

## Result

### Literature search

**Fig. 1:**
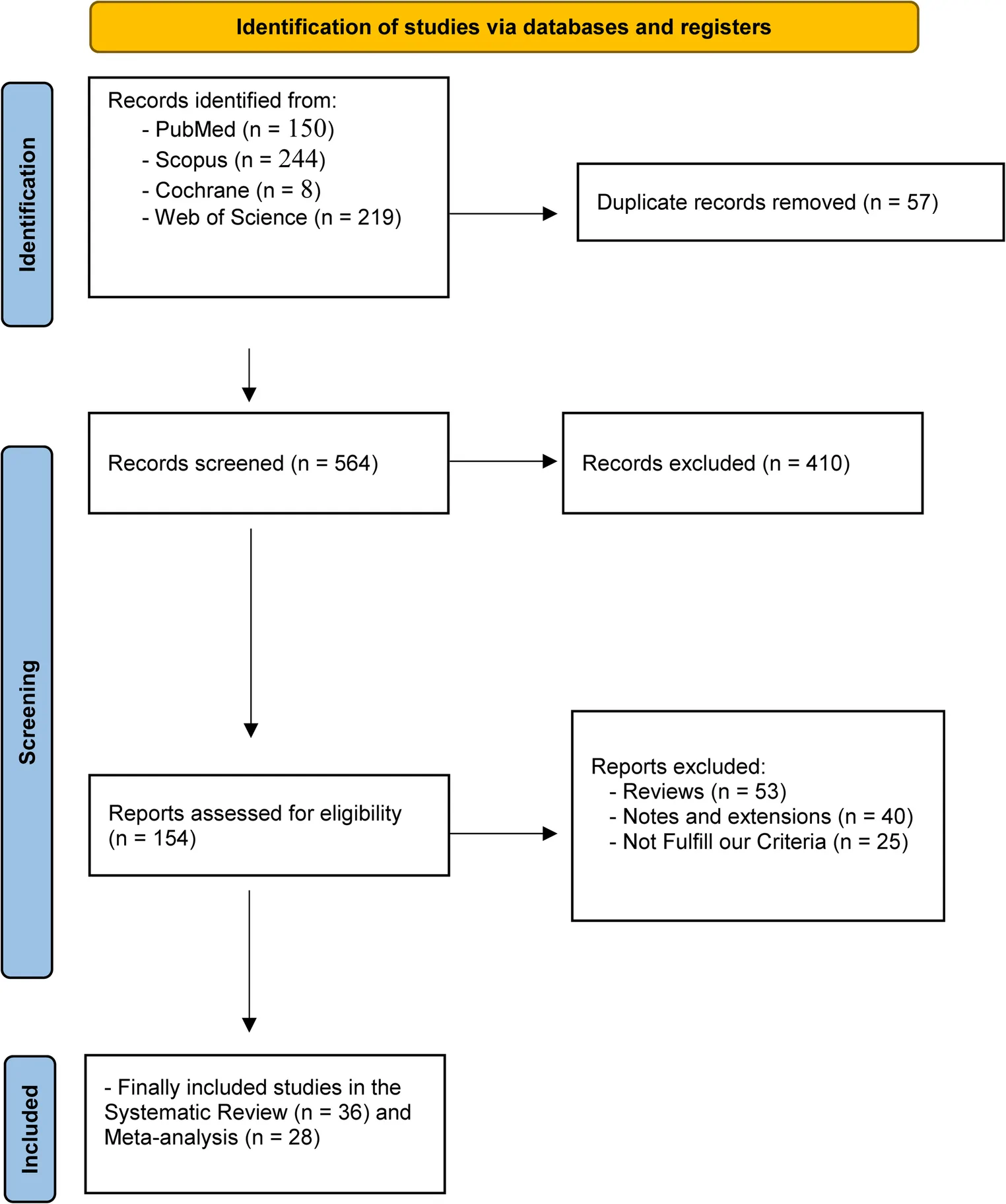
PRISMA Flow diagram

The initial database search retrieved 621 articles. 57 articles were eliminated as duplicates using EndNote. Consequently, 564 articles were available for the title and abstract screening stage. A total of 410 articles were eliminated, leaving 154 articles for the full-text review stage. 118 articles were excluded as 53 of them were review articles, 25 did not fulfill our criteria, and 40 were Notes and extensions. Finally, 36 articles were included in our primary investigation. The entire study selection process, from initial database searches to the final inclusion of studies, was documented and is presented in a PRISMA flow diagram, as shown in Fig. 1.

### Characteristics of included studies

Thirty-six studies, including 19 cohorts [4, 7, 8, 16–30], 14 case series [6, 31–43], two case-control studies [44, 45], and one cross-sectional study [46], incorporating of 491 patients were finally included in our investigation. The follow-up duration varied across the included studies, with a mean follow-up duration of 5.40 years, ranging from 0.5 to 20 years. Most of the population was of young age, with a mean age of 23.68 (1.70–65.60) years, with male gender constituting around 23% of the study participants. Detailed information concerning the summary and baseline data was presented in Table 1.

**Table 1:** Summary and baseline characteristics of included studies

Study ID	Study design	Patients assessed	Sample size	Age, y mean (SD)	Male, *n* (%)	Country	Follow-up duration	Device	Implant type	Inclusion criteria	Type of deafness	Duration of deafness	Conclusion
Alsanosi 2017	Case-control study	Usher syndrome	9	4.3 (3.2)	NA	Saudi Arabia	3 y	NA	NA	Pediatric CI recipients with genetic syndromes (including USH). Normal IQ (≥ 80, per center criteria)	Congenital sensorineural hearing loss (SNHL)	Since birth	*CIs are highly effective for USH patients, enabling near-normal auditory and speech outcomes. *Early implantation (e.g., < 2 years) and consistent rehabilitation are critical for optimal results. *Vision loss did not preclude success, but the study did not assess its impact on CI adaptation.
Aplin 1993	Retrospective cohort study	Non-Usher Deafblind Patients	5	47.4 (37.3)	3 (60)	UK	9 m	Nucleus 22-channel (*n* = 4), Ineraid (*n* = 1)	Multichannel	Postlingually deafened adults; dual sensory loss (deafblindness)	Postlingual sensorineural hearing loss	NA	*Deafblind recipients showed average-to-above-average verbal IQ (90–119). * Normal personality scores (EPQ) except one high neuroticism case. * Qualitative benefits: Reduced isolation, improved social confidence. *CI is psychologically beneficial for deafblind adults, but outcomes depend on individual adaptability.
Liu 2008	Retrospective case series	Usher syndrome	9	5.4 (4.3)	NA	USA and UK	5 y	Nucleus 22/24 M/24K, Clarion 1.2	Multichannel	USH1 diagnosis (congenital deafness + retinitis pigmentosa)	Profound sensorineural	Since birth	*CI improved auditory performance in all USH1 patients * Early implantation (≤ 3 years) showed best outcomes (60–85% open-set speech) * Genetic screening enabled early diagnosis * CI recommended before vision loss progresses
Flipo 2004	Retrospective case series	Non-Usher Deafblind Patients	6	28.7 (28.6)	2 (30.3)	Italy	NA	Clarion^®^	Multichannel	Profound deafness + blindness (Usher syndrome/rubella)	Post-lingual, Pre-lingual	NA	* Post-lingual deafblind: Achieved 100% open-set speech, restored sound localization * Pre-lingual deafblind: 65% open-set with visual cues * CI significantly improved mobility and independence
Charroó-Ruíz 2012	Prospective cohort study	Non-Usher Deafblind Patients	12	9.3 (4.2)	6 (50)	Cuba	NA	MEDICID-4 electroencephalograph	NA	NA	NA	Since birth	*Deafblind children exhibit cross-modal plasticity (tactile takeover of auditory/visual cortices). * Severity/duration of sensory loss correlates with cortical reorganization. * Supports early CI to limit interference from neuroplastic changes.
Remjasz-Jurek 2023	Retrospective cohort study	Usher syndrome	35	6.3 (4.6)	18 (51.4)	Spain	13.4 y	Cochlear™ Nucleus^®^ 22 or 24 Speech processors: Freedom or ESPrit 3G	NA	Clinical diagnosis of Usher syndrome (USH1 or USH2) based on audiovestibular/ophthalmologic exams. Congenital severe-to-profound hearing loss (HL). No inner ear anomalies on imaging (MRI/HRCT).	Profound sensorineural	Since birth	*CI significantly improves auditory performance (CAP, PTA) and speech intelligibility (SIR, MUSS) in USH children, especially if implanted before age 3. *USH patients plateaued earlier than NS children (e.g., SIR at T6 vs. T7). *ERG is critical for early USH diagnosis; vestibular dysfunction is common in USH1. *No structural inner ear anomalies were found.
Quoraishi 2020	Retrospective cohort study	Non-Usher Deafblind Patients	34	30.9 (9.3)	27 (66)	UK	4.7 y	NA	NA	Adults (≥ 18 years), Confirmed ALMS1 mutation, Audiometric data available	sensorineural hearing loss	NA	*Universal SNHL in Alström syndrome, worsening at 1.23 dB/year * 94% had deaf-blindness, necessitating early rehabilitation * CI underutilized (only 2/9 eligible received implants) *Annual audiological monitoring recommended
Dammeyer 2008	Prospective cohort study	Non-Usher Deafblind Patients	5	6.3 (1.3)	NA	Scandinavia	2.8 y	NA	NA	Congenitally deafblind children Implanted between ages 2.2–4.2 years	NA	Since birth	*Primary benefits of CI in deafblind children were non-linguistic: Improved attention, emotional response, object manipulation, and social interaction. *No significant improvement in spoken language (only 1/5 children developed intelligible speech). *Parents reported enhanced daily functioning (e.g., recognizing voices, emotional safety). *CI is a useful supplement to tactile sign language but not a replacement.
Ramsden 1993	Retrospective case series	Non-Usher Deafblind Patients	5	48 (35.5)	3 (60)	UK	19.3 y	4 patients: Nucleus 22-channel multichannel 1 patient: Single-channel extracochlear (Ineraid)	Nucleus (multichannel) Ineraid (single-channel)	Postlingually deafblind adults, Profound deafness (no benefit from hearing aids), Willingness to undergo rehabilitation	Postlingual	14 y	*3/4 multichannel users achieved open-set speech discrimination (telephone use with familiar voices). *1/5 discontinued use due to tinnitus exacerbation. *Single-channel implant provided only environmental sound awareness (no speech perception).
Saeed 1998	Retrospective case series	Non-Usher Deafblind Patients	10	43.6 (24.4)	9 (90)	UK	6 y	9 patients: Nucleus C122M (multichannel) 1 patient: UCH/RNID (single-channel)	Nucleus C122M (multichannel) UCH/RNID (single-channel)	Severe/profound deafness (no benefit from hearing aids), Blind or visually impaired, Willingness to undergo rehabilitation	NA	NA	*3/7 adults with multichannel implants achieved exceptional BKB scores (≥ 94%), surpassing sighted implant averages. *Postlingual deaf-blind adults performed best (e.g., Case 1: 98% BKB at 18 months). *Children (Usher’s/congenital rubella): Demonstrated auditory awareness (Ling sounds) but slower progress. *Single-channel implant: Provided only environmental sound awareness (no speech perception).
Hoshino 2016	Retrospective case series	Usher syndrome	10	18.9 (14.6)	7 (70)	Brazil	1 y	Cochlear Nucleus (24 M/K, Freedom) Neurolec Digisonic SP Medel Sonata AB HiRes 90k	Multichannel	Confirmed Usher Syndrome Type I (USH1), Prelingual deafness, Progressive vision loss (retinitis pigmentosa), Late cochlear implantation (≥ 5 years old)	Prelingual profound SNHL	Since birth	*Sound detection improved (PTA: 103 dB HL → 35 dB HL). *Limited speech recognition: Only 3/10 achieved closed-set sentence understanding (10–40%). *30% abandonment rate (3/10) due to lack of benefit.
Loundon 2003	Retrospective cohort study	Usher syndrome	13	1.7 (1.5)	NA	France	4.1 y	Nucleus CI22, CI24, Clarion HF	NA	Congenital profound deafness + Usher syndrome (diagnosed via ERG, fundoscopy, vestibular testing)	Profound sensorineural hearing loss	Since birth	*Early CI in Usher syndrome improves speech perception/production, especially if implanted before age 9. * Delayed walking (> 17 months) + deafness should prompt ERG for early diagnosis. * Vestibular dysfunction common (92.3%).
Daneshi 2006	Retrospective cohort study	Non-Usher Deafblind Patients	3	5.7 (2.1)	2 (66.7)	Iran	1 y	Med-El combi40 + or Nucleus CI22/CI24	NA	Prelingually deaf children with additional disabilities (congenital blindness, autism, cerebral palsy, etc.)	Profound prelingual deafness	Since birth	Limited auditory improvement post-CI (mean score: 1.0 pre-CI → 15.0 post-CI, *p* = 0.102). Deafblind children require unique rehabilitation strategies due to poor outcomes compared to other disabilities.
Green 2008	Retrospective case series	Non-Usher Deafblind Patients	2	48.5 (5.5)	2 (100)	UK	13 y	NA	NA	Congenitally blind + post-lingually deaf adults with Cis	Post-lingual profound deafness	1 y	*Patient 1 (short deafness): Auditory cortex activation (+ 9.34% L). *Patient 2 (longer deafness): Visual cortex dominance (+ 10.15% R) for auditory processing. *Supports cross-modal plasticity in deafblind CI users, with visual cortex compensating for auditory input.
Henricson 2012	Cross-sectional study	Usher syndrome	7	10.3 (3)	NA	Sweden	NA	NA	NA	Confirmed diagnosis of Usher Syndrome Type 1 (USH1) (genetically or clinically), Age 6–16 years.	Congenital, profound bilateral deafness	Since birth	*Children with USH1 + CI performed similarly to hearing-impaired children with hearing aids (HI using HA) in most cognitive tasks. *They outperformed non-USH1 deaf children with CI in phonological working memory and lexical skills, possibly due to better auditory processing or early diagnosis. *Early implantation (< 2 years) correlated with better cognitive outcomes.
Takano 2015	Retrospective cohort study	Non-Usher Deafblind Patients	8	65.6 (8.9)	2 (25)	Japan	20 y	Nucleus C124RE, C122M, COMBI40, N22, M24, C124R(CS), C124M	NA	Severe-to-profound sensorineural hearing loss, Significant visual impairment, Postlingual deafness	profound sensorineural hearing loss (SNHL)	1 y	*Speech Perception: Deafblind patients achieved comparable scores to non-deafblind CI users (72.3% words, 86.0% sentences on CI-2004 test). *Communication: 7/8 transitioned from tactile methods (finger braille, bold letters) to voice conversations; 5/8 could use a telephone. *Challenges: 1 patient struggled in noisy environments.
Iwasaki 2017	Retrospective cohort study	Usher syndrome	19	47 (19)	7 (37)	Finland	6 y	MED-EL, and Nucleus	NA	Confirmed USH3 diagnosis (genetically and clinically), Progressive sensorineural hearing loss (SNHL) + retinitis pigmentosa (RP), Previous hearing aid use.	Progressive severe-to-profound SNHL	30 y	*CI significantly improved hearing (mean PTA: 34 ± 9 dB HL post-CI vs. 58 ± 11 dB HL with hearing aids, *p*<0.001). *Speech recognition improved (52 ± 33% post-CI vs. 4 ± 9% pre-CI, *p*<0.01). *Quality of life (QoL) benefits (GBI score: 30 ± 19; comparable to non-deafblind CI users). *High compliance: 88% used CI > 12 h/day; 95% required no assistance.
Nair 2019	Retrospective cohort study	Usher syndrome	27	2.9 (1.8)	18 (66.7)	India	1 y	NA	NA	Age 1–6 years, Confirmed Usher Syndrome (congenital SNHL + retinitis pigmentosa), Normal cochlear anatomy.	profound sensorineural hearing loss (SNHL)	NA	*Auditory Improvement: Significant but slower progress in CAP/SIR scores vs. non-syndromic peers (*p* < 0.05). -CAP: 4.4/7 at 12 months (vs. 5.2 in controls). -SIR: 3.0/5 at 12 months (vs. 4.3 in controls). *Quality of Life: Lower QoL scores (GBI: 9/18; HUI 3.0: 17/40) vs. controls (*p* < 0.05). *Clinical Implications: -Requires intensive, individualized therapy due to dual sensory loss. -Early CI (< 6 years) critical to optimize outcomes before vision deteriorates.
Teschner 2007	Retrospective cohort study	Usher syndrome	117	NA	NA	Germany	NA	NA	NA	Congenitally deaf children (100% hearing loss per Roser criteria)	Profound sensorineural hearing loss	Since birth	*Minimized Rotation vestibular testing identified 16.2% of deaf children with vestibular areflexia; 50% of these (8.1% of total cohort) had retinal dysfunction (Usher Type I). *Early bilateral CI is recommended for Usher Type I to maximize auditory rehabilitation before blindness progresses.
Jatana 2013	Retrospective cohort study	Usher syndrome	26	3.3 (2.6)	17 (65.4)	USA	7.8 y	NA	NA	Children with Usher Syndrome Type I (congenital profound SNHL + RP), Confirmed diagnosis by electroretinography (ERG) and/or genetic testing	Congenital profound sensorineural hearing loss	Since birth	*Bilateral CI recipients (100%) were more likely to use oral communication than unilateral (42.9%) *Early bilateral CI recommended to maximize auditory rehabilitation before vision loss progresses *Delayed ambulation (mean 21.9 months) and ERG are key for early USH diagnosis
Nishizaki 1999	Retrospective case series	Non-Usher Deafblind Patients	2	35.6 (5.6)	2 (100)	Japan	2 y	Nucleus Mini-22	NA	Progressive hereditary deafness, Associated visual impairment (albinism/CPEO), Positive promontory stimulation test, Normal cochlear anatomy on imaging	sensorineural hearing loss	NA	*Both patients showed significant improvement in speech perception: -Vowel discrimination: 80–95% (Case 1), 75–100% (Case 2) -Consonant discrimination: 61–75% (Case 1), 43–79% (Case 2) *Transitioned from writing-only to oral communication *Telephone use achieved in both cases
Daneshi 2022	Retrospective cohort study	Non-Usher Deafblind Patients	29	20.5 (21.4)	19 (65.6)	Iran and USA	2 y	NA	NA	Bilateral severe-to-profound SNHL, Complete electrode insertion, Regular auditory rehabilitation, Blindness (WHO Classes 3–5)	NA	NA	*Auditory Performance (CAP): -No significant difference between deaf-blind and deaf-only children. -70% reached CAP 6–7 (“conversation understanding/telephone use”). *Speech Intelligibility (SIR): -Deaf-blind children scored lower than deaf-only (*p*<0.01). -80% achieved SIR 4–5 (“intelligible to unfamiliar listeners”).
Ricci 2014	Retrospective case series	Non-Usher Deafblind Patients	5	3 (1.4)	3 (60)	Italy	2.5 y	NA	Multi-electrode arrays	CHARGE syndrome diagnosis, Bilateral profound SNHL, No cochlear nerve aplasia	Congenital profound sensorineural hearing loss	Since birth	Surgical Feasibility: CI achievable despite complex anatomy (2 via mastoidotomy, 3 via suprameatal approach)
Damen 2006	Retrospective cohort study	Usher syndrome	14	26.4 (5.6)	9 (64)	Netherlands	9.2 y	Nucleus 22, 24	NA	Clinical diagnosis of Usher Syndrome Type I (USH1) (congenital profound deafness + retinitis pigmentosa + vestibular areflexia). CI users had implantation at the Nijmegen-St. Michielsgestel center.	Congenital profound sensorineural hearing loss	Since birth	*CI significantly improved hearing-related QoL (NCIQ scores) in USH1 patients, particularly in sound perception. *No improvement in generic QoL (SF12). *CI users required less assistance for independence (communication, mobility) compared to non-CI deaf-blind patients.
Chute 1995	Retrospective case series	Usher syndrome	3	NA	1 (33.3)	USA	3 y	Nucleus 22-channel	NA	Profound bilateral sensorineural hearing loss, No benefit from conventional amplification, Diagnosis of Usher syndrome	Congenital profound sensorineural hearing loss	Since birth	*CI improved auditory perception in all 3 children * Younger age at implantation correlated with better outcomes * Improved environmental awareness and mobility
Raine 2008	Retrospective case series	Non-Usher Deafblind Patients	2	64 (1.2)	0 (0)	UK	2 y	Nucleus CI24 (Cochlear Ltd)	NA	Refsum’s disease with profound sensorineural hearing loss and retinitis pigmentosa (blindness)	Sensorineural hearing loss	NA	Cochlear implantation significantly improved speech perception, sound localization (bilateral case), and quality of life in deafblind patients with Refsum’s disease. Bilateral implantation is beneficial for dual sensory loss.
Soper 2006	Retrospective case series	Non-Usher Deafblind Patients	5	39 (20.2)	5 (100)	UK	NA	NA	NA	Adults with acquired deafblindness (post-lingual hearing loss + progressive visual impairment)	Sensorineural hearing loss	Since birth	Cochlear implants improved communication (speech recognition, lip-reading) and quality of life (reduced isolation) but had limited impact on mobility (sound localization remained poor). Benefits were greatest for post-lingually deafened adults. Bilateral implantation was suggested for future research.
El-Kashlan 2001	Retrospective case series	Non-Usher Deafblind Patients	8	33.4 (24.4)	4 (50)	USA	6 m − 9 y	Nucleus 22, Clarion, Nucleus CT24M	Multichannel cochlear implants	Profound SNHL + significant visual impairment (legal blindness or progressive vision loss)	Sensorineural hearing loss	NA	Cochlear implants significantly improved speech perception (CID scores up to 100%), communication, and QoL in deafblind patients, especially post-lingually deafened adults. Deafblind patients outperformed sighted CI recipients in speech scores (83% vs. 53%), suggesting cross-modal cortical plasticity. Rehabilitation requires specialized tactile communication training.
Hinderink 2015	Retrospective cohort study	Non-Usher Deafblind Patients	4	21.2 (9.8)	2 (50)	Netherlands	2 y	Single-channel (Med-EI E/1 or 3 M/Vienna) Multichannel (Nucleus 22)	2 Usher’s patients: Single-channel 2 Usher’s patients: Multichannel	Congenital profound deafness (PTA > 110 dB HL), Diagnosis of Usher syndrome type 1 (deafness + retinitis pigmentosa), No additional mental/sensory deficits, Ability to lip-read and use oral-aural communication	Prelingual sensorineural hearing loss (SNHL)	Since birth	*Cochlear implantation improved suprasegmental speech perception and environmental sound awareness in deafblind (Usher’s) patients, though no open-set speech recognition was achieved. *No significant difference in outcomes between single- and multichannel implants. *Subjective benefits included enhanced safety, social integration, and reduced isolation. *Rehabilitation required minimal extra effort compared to non-deafblind prelingually deaf patients.
Wiley 2013	Retrospective cohort study	Non-Usher Deafblind Patients	12	4.5 (0.9)	NA	USA	3.5 y	NA	Multichannel	Children aged 6 months–8 years with dual sensory impairment (deafblindness). Totally blind (no light perception) or light perception only (combined in analysis).	NA	Since birth	*Receptive Language: - 16.7% reached “understands functional use of objects” (highest level). - 58.3% achieved only sound detection or simple word responses. *Expressive Language: - 0% achieved complex sentences (vs. 12% in total cohort). - 41.7% communicated with sound production only.
Ahn 2013	Retrospective case series	Non-Usher Deafblind Patients	6	4.9 (3.3)	4 (66.7)	South Korea	4.9 y	Cochlear™ models (CI512, CI24R, CI24RE, CI24RST)	NA	Pediatric patients with CHARGE syndrome and SNHL. Confirmed inner ear anomalies on imaging.	Sensorineural hearing loss (SNHL)	Since birth	*CI improved auditory outcomes in most CHARGE patients, including those with developmental delays. *No direct correlation between anatomic anomalies (e.g., coloboma) and CI outcomes. *Blindness-specific outcomes were not reported (only 1 patient had coloboma, and visual status was not detailed).
Birman 2015	Retrospective cohort study	Non-Usher Deafblind Patients	10	9.8 (4.3)	4 (40)	Australia	12 y	Cochlear™ (unspecified model)	NA	CHARGE syndrome + CI candidates	Sensorineural hearing loss (SNHL)	Since birth	Children with CHARGE + congenital deafness had poor verbal outcomes; progressive SNHL group maintained speech. Visual status not directly linked to CI outcomes.
Broomfield 2013	Retrospective cohort study	Usher syndrome	9	10.4 (3.2)	NA	UK	8 y	NA	NA	Genetic confirmation + CI candidacy	NA	Since birth	Variable outcomes: 4/9 achieved open-set speech (SRS = 6); older children with progressive vision loss relied on sign language. Early CI correlated with better speech outcomes.
Mesallam 2019	Case-control study	Usher syndrome	6	9.4 (2.5)	4 (66.7)	Saudi Arabia	1 y	NA	NA	Pre-lingual severe-to-profound sensorineural hearing loss. Diagnosis of Usher syndrome (subtype I, congenital deafness + progressive retinitis pigmentosa). CI used for ≥ 12 months. ≥12 months of auditory-verbal therapy post-CI.	Congenital sensorineural hearing loss	Since birth	*Usher syndrome children showed comparable auditory (MAIS) and speech (MUSS) outcomes to non-disabled CI recipients. *4/6 (66.7%) progressed from behavioral to oral communication post-CI. *Early CI is critical for optimal outcomes, as visual decline later may hinder rehabilitation.
Young 2017	Retrospective cohort study	Non-Usher Deafblind Patients	12	3.5 (2.1)	7 (58.3)	USA	4.7 y	Cochlear Americas Contour Advance, Advanced Bionics UJ and Mid-scala electrode arrays	NA	Children with CHARGE syndrome who received a cochlear implant and had at least 12 months of implant use	Sensorineural hearing loss (SNHL)	Since birth	*Most children with CHARGE syndrome achieved at least improved detection with cochlear implants (CI). *Half of the children acquired some level of speech perception, though progress was slower compared to neurotypical deaf children. *Cochlear nerve deficiency (CND) was common but should not preclude CI candidacy. *Parental perception of benefit was positive for most children, highlighting the importance of CI for quality of life and communication.
Chen 2025	Retrospective case series	Usher syndrome	2	4.3 (1.1)	1 (50)	China	0.9 y	Synergy 2	NA	Congenital SNHL with PCDH15 mutations	Profound sensorineural hearing loss	Since birth	CI improved auditory outcomes in USH1F patients. Early implantation (≤ 2y) correlated with excellent speech recognition (CAP 6–7). Retinal degeneration requires long-term monitoring.

Abbreviations: *CI* Cochlear implant, *SNHL* Sensorineural hearing loss, *USH* Usher syndrome, *ALMS* Alström syndrome, *CHARGE* Coloboma, Heart defects, Atresia choanae, Retarded growth, Genital anomalies, Ear anomalies, *PTA* Pure-tone average, *SIR* Speech Intelligibility Rating, *CAP* Categories of Auditory Performance, *MAIS* Meaningful Auditory Integration Scale, *MUSS* Meaningful Use of Speech Scale, *BKB* Bamford–Kowal–Bench sentence test, *EPQ* Eysenck Personality Questionnaire, ERG Electroretinography, *QoL* Quality of life, *HA* Hearing aid, *HL* Hearing loss

### Risk of bias assessment

Eighteen studies were assessed by the NIH tool for single-arm, of which 11 were rated as good quality, and 7 were rated as Fair quality. Detailed information is in Table 2. Fourteen studies were evaluated by the NIH tool for case series, of which 2 were rated as good quality, 8 were rated as Fair quality, and 4 were rated as poor quality. Four studies were evaluated using the NOS tool, of which two were rated as high quality and two as low quality. A detailed summary of the risk of bias assessment for each study is presented in Supplementary Tables 2 and 3.

**Table 2:** NIH Quality assessment for single-arm studies

Study ID	Was the research question or objective in this paper clearly stated?	Were eligibility/selection criteria for the study population prespecified and clearly described?	Were the participants in the study representative of those who would be eligible for the test/service/intervention in the general or clinical population of interest?	Were all eligible participants that met the prespecified entry criteria enrolled?	Was the sample size sufficiently large to provide confidence in the findings?	Was the test/service/intervention clearly described and delivered consistently across the study population?	Were the outcome measures prespecified, clearly defined, valid, reliable, and assessed consistently across all study participants?	Were the people assessing the outcomes blinded to the participants’ exposures/interventions?	Was the loss to follow-up after baseline 20% or less? Were those lost to follow-up accounted for in the analysis	Were outcome measures of interest taken multiple times before the intervention and multiple times after the intervention (i.e., did they use an interrupted time-series design)?	If the intervention was conducted at a group level (e.g., a whole hospital, a community, etc.) did the statistical analysis take into account the use of individual-level data to determine effects at the group level?	Did the statistical methods examine changes in outcome measures from before to after the intervention? Were statistical tests done that provided *p* values for the pre-to-post changes?	Final score	Final assemseent Quality Rating: Poor < 50%, Fair 50–75%, Good ≥ 75%.
Alsanosi 2017	yes	yes	yes	No	No	yes	yes	No	yes	No	yes	yes	66%	Fair Quality
Aplin 1993	yes	yes	yes	No	No	yes	yes	No	yes	No	yes	yes	66%	Fair Quality
Charroó-Ruíz 2012	yes	yes	yes	No	yes	yes	yes	No	yes	No	yes	yes	75%	Good Quality
Quoraishi 2020	yes	yes	yes	No	No	yes	yes	No	yes	No	yes	yes	66%	Fair Quality
Remjasz-Jurek 2023	yes	yes	yes	No	yes	yes	yes	No	yes	No	yes	yes	75%	Good Quality
Loundon 2003	yes	yes	yes	No	No	yes	yes	No	yes	No	yes	yes	66%	Fair Quality
Daneshi 2006	yes	yes	yes	No	No	yes	yes	No	yes	No	yes	yes	66%	Fair Quality
Henricson 2012	yes	yes	yes	No	No	yes	No	No	yes	No	yes	yes	58%	Fair Quality
Takano 2015	yes	yes	yes	No	yes	yes	yes	No	yes	No	yes	yes	75%	Good Quality
Iwasaki 2017	yes	yes	yes	No	yes	yes	yes	No	yes	No	yes	yes	75%	Good Quality
Teschner 2007	yes	yes	yes	No	No	yes	No	No	yes	No	yes	yes	58%	Fair Quality
Jatana 2013	yes	yes	yes	No	yes	yes	yes	No	yes	No	yes	yes	75%	Good Quality
Daneshi 2022	yes	yes	yes	No	yes	yes	yes	No	yes	No	yes	yes	75%	Good Quality
Ricci 2014	yes	yes	yes	No	yes	yes	yes	No	yes	No	yes	yes	75%	Good Quality
Damen 2006	yes	yes	yes	No	yes	yes	yes	No	yes	No	yes	yes	75%	Good Quality
Birman 2015	yes	yes	yes	No	yes	yes	yes	No	yes	No	yes	yes	75%	Good Quality
Broomfield 2013	yes	yes	yes	No	yes	yes	yes	No	yes	No	yes	yes	75%	Good Quality
Young 2017	yes	yes	yes	No	yes	yes	yes	No	yes	No	yes	yes	75%	Good Quality

## Results

### Primary clinical outcome

#### Speech & communication outcomes

##### **Speech Intelligibility Rating (SIR) Scale score**

**Fig. 2:**
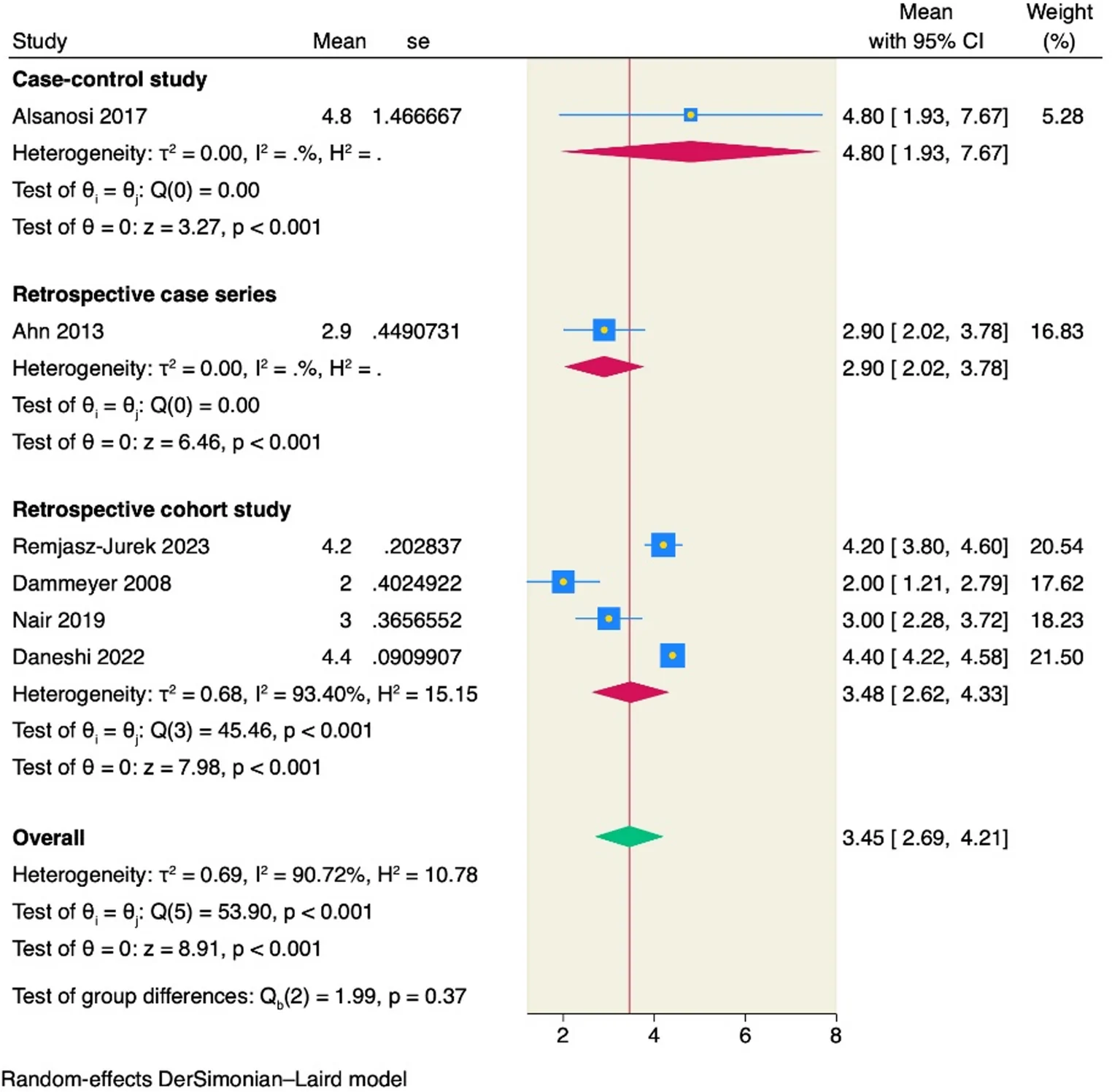
Speech Intelligibility Rating (SIR) Scale score with subgroup on study design

**Fig. 3:**
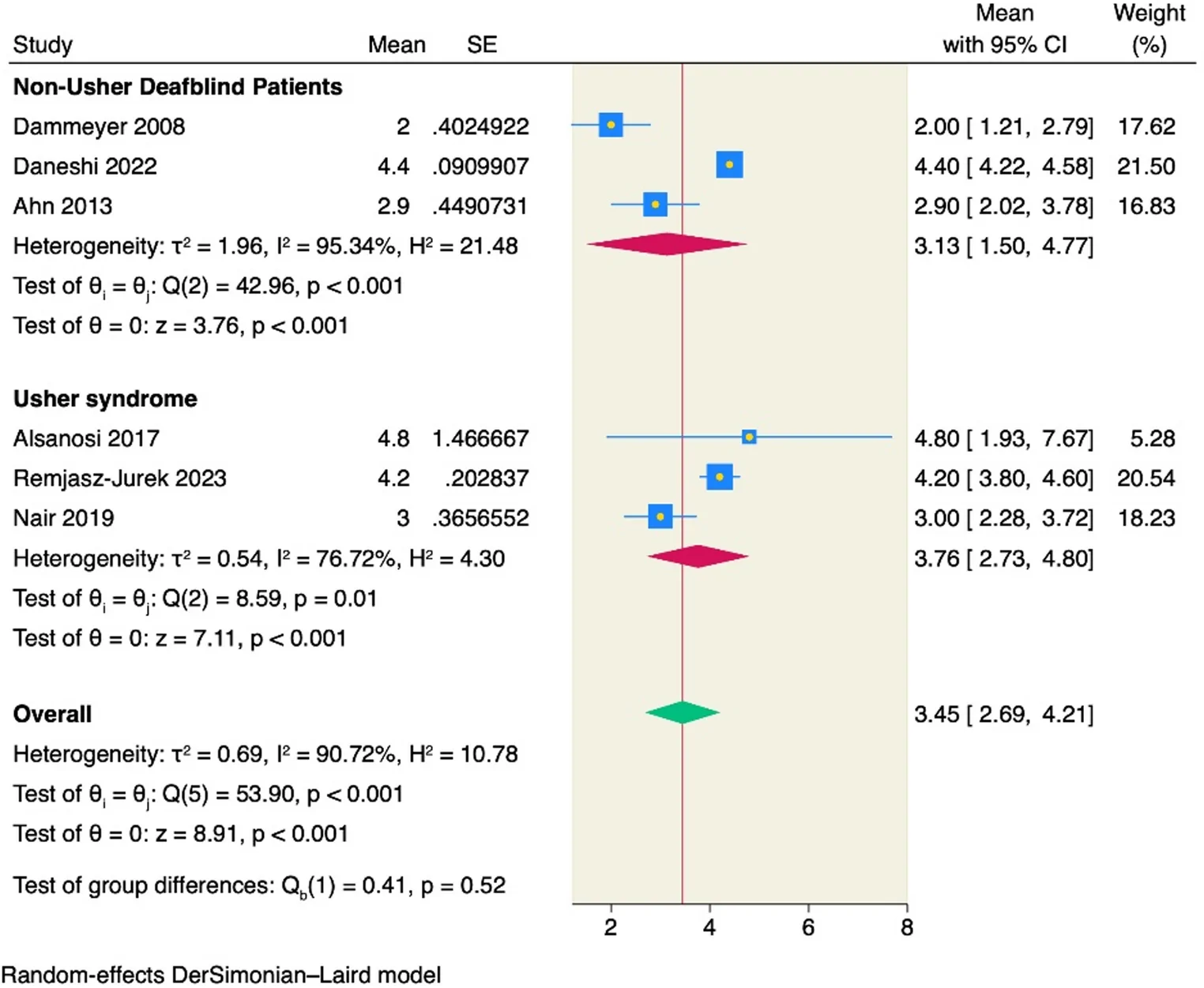
Speech Intelligibility Rating (SIR) Scale score with subgroup on Usher and non-Usher

Pooled analysis of the SIR scale using a random-effects DerSimonian–Laird model demonstrated a clinically meaningful improvement in speech intelligibility after implantation, with a pooled mean SIR score of 3.45, 95% CI: 2.69 to 4.21, p < 0.001. A subgroup analysis stratified by study design showed that improvements in speech intelligibility were consistently observed across all study designs, with pooled mean SIR scores of 4.80 (95% CI: 1.93 to 7.67, p < 0.001) in case–control studies, 2.90 (95% CI: 2.02 to 3.78, p < 0.001) in retrospective case series, and 3.48 (95% CI: 2.62 to 4.33, p < 0.001) in retrospective cohort studies. No statistically significant differences were detected between study design subgroups (Qb = 1.99, p = 0.37) (Fig. 2). Further subgroup analysis based on syndromic status demonstrated that speech intelligibility improvement following CI was evident in both groups, with pooled mean SIR scores of 3.76 (95% CI: 2.73 to 4.80, p < 0.001) in patients with Usher syndrome and 3.13 (95% CI: 1.50 to 4.77, p < 0.001) in non-Usher deafblind patients, with no statistically significant subgroup effect (Qb = 0.41, p = 0.52) (Fig. 3). The Galbraith plot showed that most studies clustered within the confidence limits, suggesting the absence of major outlier studies. However, substantial statistical heterogeneity remained across studies, as indicated by the high I² value. In contrast, the funnel plot displayed some asymmetry, indicating a possible risk of publication bias (Supplementary Figs. 1–2).

##### **Verbal Communication Ability**

**Fig. 4:**
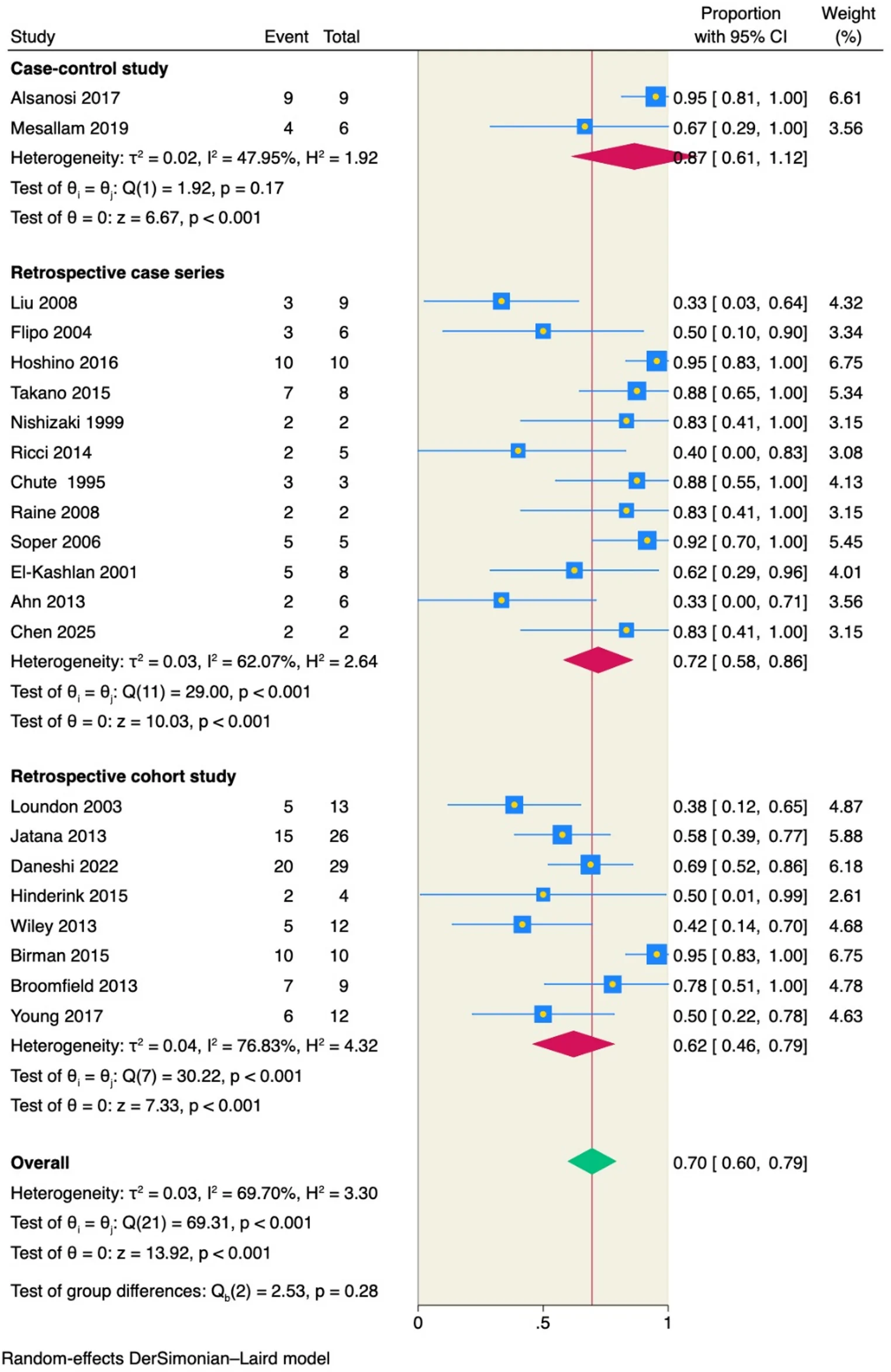
Verbal Communication Ability with the subgroup on study design

**Fig. 5:**
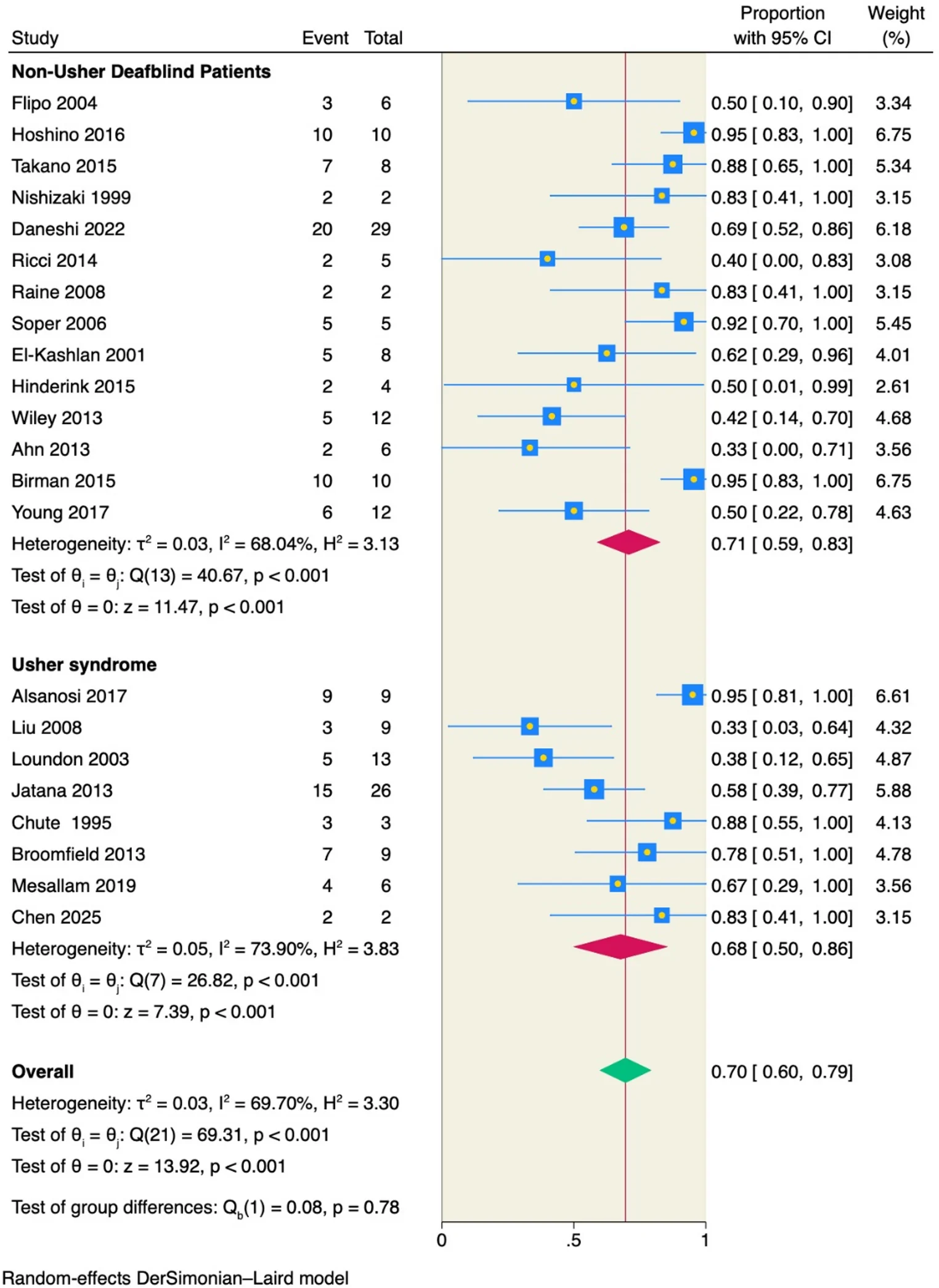
Verbal Communication Ability with Subgroup on Usher and Non-Usher

Pooled analysis of verbal communication ability using a random-effects DerSimonian–Laird model demonstrated a clinically meaningful likelihood of achieving functional verbal communication following cochlear implantation, with an overall pooled proportion of 0.70 (95% CI: 0.60 to 0.79; p < 0.001). Subgroup analysis stratified by study design showed consistent and favorable outcomes across all study types, with pooled proportions of 0.87 (95% CI: 0.61 to 1.12, p < 0.001) in case–control studies, 0.72 (95% CI: 0.58 to 0.86, p < 0.001) in retrospective case series, and 0.62 (95% CI: 0.46 to 0.79, p < 0.001) in retrospective cohort studies. No statistically significant differences were observed between study design subgroups (Qb = 2.53, p = 0.28) (Fig. 4). Further subgroup analysis based on syndromic status demonstrated significant improvement in verbal communication outcomes following cochlear implantation, with comparable rates between groups. The pooled proportion was 0.68 (95% CI: 0.50–0.86) in patients with Usher syndrome and 0.71 (95% CI: 0.59–0.83) in non-Usher deafblind patients, with no statistically significant subgroup effect observed (Qb = 0.08, p = 0.78) (Fig. 5). The Galbraith plot showed that most studies clustered within the 95% precision limits, suggesting consistency across studies. In contrast, the funnel plot demonstrated asymmetry, indicating a potential risk of publication bias (Supplementary Figs. 3–4).

##### **Telephone Communication Competence**

**Fig. 6:**
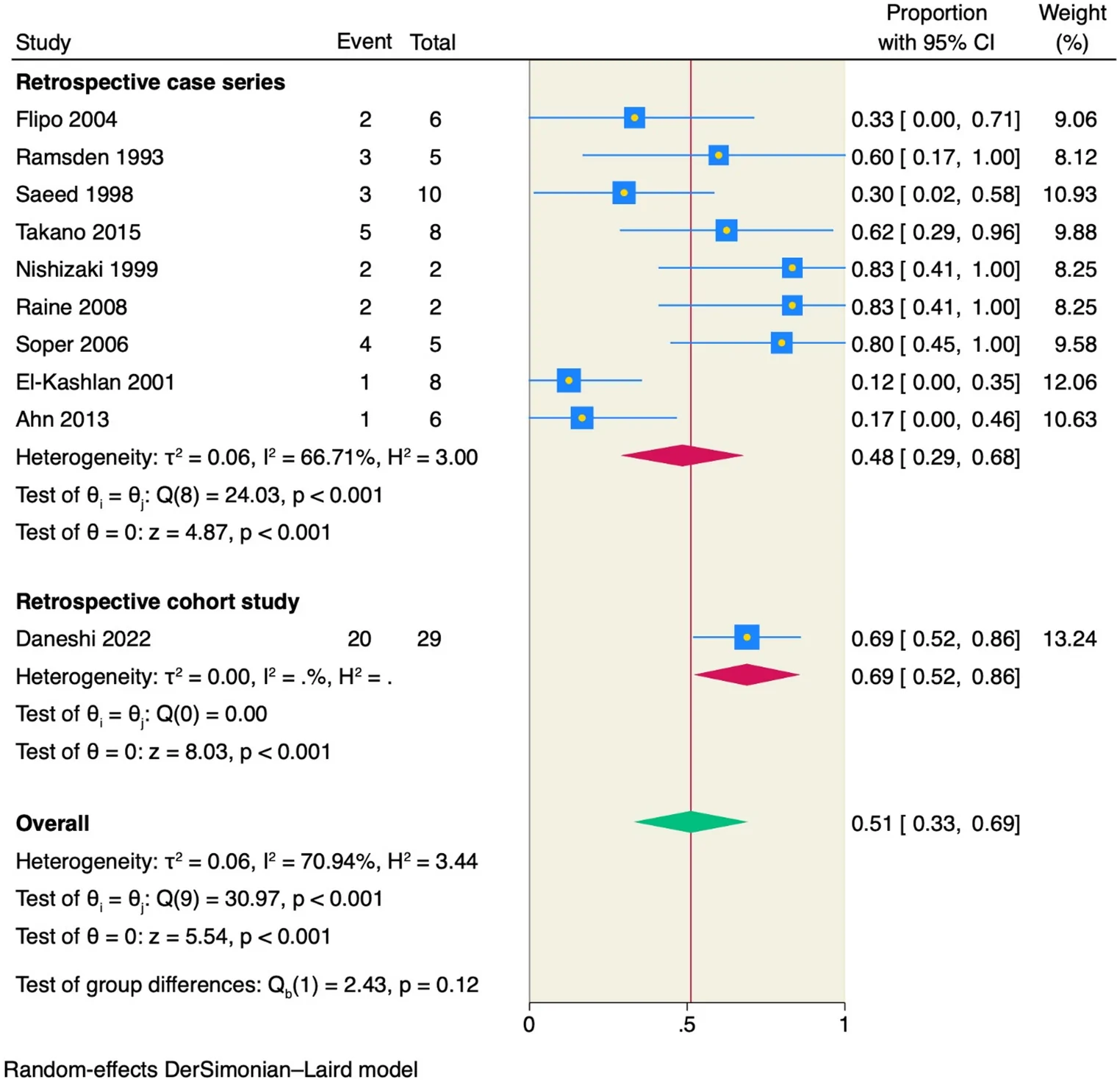
Telephone communication competence with the subgroup on the study design

Pooled analysis of telephone communication competence using a random-effects DerSimonian–Laird model demonstrated a clinically meaningful likelihood of achieving functional telephone communication following cochlear implantation, with an overall pooled proportion of 0.51 (95% CI: 0.33 to 0.69; p < 0.001). Subgroup analysis stratified by study design showed generally favorable outcomes, with a pooled proportion of 0.48 (95% CI: 0.29 to 0.68, p < 0.001) in retrospective case series and 0.69 (95% CI: 0.52 to 0.86, p < 0.001) in retrospective cohort studies. No statistically significant differences were observed between study design subgroups (Qb = 2.43, p = 0.12) (Fig. 6). The Galbraith plot showed that most studies clustered within the 95% precision limits, suggesting consistency across studies despite moderate heterogeneity. In contrast, the funnel plot demonstrated asymmetry, indicating a potential risk of publication bias (Supplementary Figs. 5–6).

#### Secondary outcomes

##### **Audiological Performance Outcomes**

Pooled analysis using a random-effects DerSimonian–Laird model demonstrated a significant improvement in post-implant pure-tone average (PTA), with an overall mean of 32.4 dB HL (95% CI: 25.4 to 39.4; p < 0.001). Subgroup analysis by syndromic status showed significant post-implant PTA improvement in both groups, with higher PTA values observed in non-Usher deafblind patients compared with those with Usher syndrome; however, the between-group difference was not statistically significant (Qb = 2.35, p = 0.13) (Supplementary Fig. 7). Similarly, random-effects meta-analysis demonstrated a significant improvement in word recognition following implantation, with a pooled mean score of 62.3% (95% CI: 45.8 to 78.8; p < 0.001), with no observed heterogeneity (I² = 0%) (Supplementary Fig. 8). Additionally, random-effects meta-analysis showed a significant likelihood of successful environmental sound detection after implantation (pooled proportion: 0.61; 95% CI: 0.41–0.82; p < 0.001), with retrospective cohort studies demonstrating higher detection rates than case series. Although the difference between study-design subgroups was not statistically significant (Qb = 3.32, p = 0.07) (Supplementary Fig. 9).

##### **Auditory & Cognitive Development Outcomes**

Random-effects meta-analysis demonstrated a significant improvement in auditory performance following implantation, with an overall pooled mean CAP score of 4.59 (95% CI: 3.62 to 5.56; p < 0.001). Subgroup analyses by study design and syndromic status showed consistent improvements across all subgroups, with no statistically significant differences between subgroups (Supplementary Figs. 10–11). Age-appropriate cognitive skills were reported in a high proportion of patients, with a pooled estimate of 93% (95% CI: 0.82 to 1.04). However, most included studies did not report pre-implant cognitive baseline assessments or appropriate control groups. Therefore, these findings should be interpreted cautiously and cannot establish a causal relationship between cochlear implantation and cognitive improvement. (Supplementary Fig. 12).

##### **Educational & Social Adaptation**

Random-effects meta-analysis demonstrated a statistically significant likelihood of successful educational integration following implantation, with an overall pooled proportion of 0.36 (95% CI: 0.05 to 0.68; p = 0.02). Substantial between-study heterogeneity was observed (I² = 73.9%) (Supplementary Fig. 13).

##### **Quality of Life & Psychosocial Benefits**

Random-effects meta-analysis demonstrated a significant positive impact of family and social support following implantation, with an overall pooled proportion of 0.67 (95% CI: 0.37 to 0.96; p < 0.001). Low between-study heterogeneity was observed (I² = 18.15%) (Supplementary Fig. 14).

##### **Advanced Auditory & Musical Skills**

Musical perception was achieved in a minority of patients following cochlear implantation, with a statistically significant pooled proportion of 19% (95% CI: 0.03–0.35) able to play the piano after implantation (Supplementary Fig. 15). In contrast, daily radio listening was reported in 14% of patients, with no statistically significant effect observed (95% CI: −0.02 to 0.30) (Supplementary Fig. 16).

## Discussion

Deafblindness, whether syndromic (e.g., Usher syndrome, CHARGE syndrome) or non-syndromic, presents a profound sensory deprivation that significantly impairs communication, social integration, and quality of life. Historically, individuals with dual sensory loss were often excluded from cochlear implantation (CI) programs due to concerns about limited auditory rehabilitation outcomes [44]. However, advancements in CI technology, surgical techniques, and post-implantation rehabilitation have expanded candidacy criteria, prompting a reevaluation of CI’s role in this population [4].

Despite these advancements, research on CI outcomes in deafblind individuals remains limited, with existing studies often characterized by small sample sizes, heterogeneous populations, and variable outcome measures [6]. This systematic review and meta-analysis synthesize evidence from 36 studies including 491 patients, providing the most comprehensive summary to date of CI outcomes in this population. Our findings not only validate the benefits of CI in this population but also highlight areas requiring further investigation to optimize patient selection and post-implantation support.

The pooled mean Speech Intelligibility Rating (SIR) score of 3.45 demonstrates that CI can significantly improve speech clarity in deafblind individuals, with no notable differences between Usher syndrome and non-Usher patients (*p* = 0.78). This suggests that the presence of visual impairment does not preclude successful auditory rehabilitation, reinforcing the potential of CI to restore functional communication. Verbal communication was achieved in 70% of patients, a finding consistent with prior studies in non-deafblind CI recipients. These rates are consistent with reported verbal communication outcomes in CI users with additional disabilities and in general CI cohorts, indicating that the absence of visual cues does not preclude the development of spoken language when adequate auditory input is provided [6, 47].

However, telephone communication competence remained limited, with only half of the patients demonstrating success. Notably, similar challenges with telephone use have also been reported among cochlear implant recipients without visual impairment, reflecting the auditory complexity of telephone communication and reduced acoustic cues. While the absence of visual feedback may further increase these challenges in deafblind individuals, the difficulty cannot be attributed solely to visual impairment [48]. These results underscore the need for tailored rehabilitation strategies that incorporate multisensory compensatory techniques to enhance telephone use and other complex auditory tasks.

Our analysis revealed a mean pure-tone average (PTA) of approximately 32 dB HL is comparable to thresholds typically reported in non-deafblind adult and pediatric CI populations, indicating that peripheral auditory benefit from CI is preserved despite concomitant visual impairment. Subgroup analysis showed a trend toward better outcomes in Usher syndrome patients (29.28 dB) compared to non-Usher cases (38.1 dB), possibly due to earlier intervention before progressive vision loss complicates auditory adaptation. Word recognition (62.3%) and environmental sound detection (61%) further support CI’s efficacy, though the high heterogeneity in environmental sound outcomes (I² = 76.53%) suggests variability in assessment methods or patient-specific factors.

A striking 93% of patients achieved age-appropriate cognitive skills post-CI, highlighting the critical role of auditory input in neurodevelopment, particularly in preventing the cognitive delays associated with sensory deprivation [48]. The mean Categories of Auditory Performance (CAP) score of 4.59 reflects intermediate-to-advanced auditory processing, which is essential for social and educational integration. Despite these gains, only 36% of patients were enrolled in mainstream schooling, indicating persistent systemic barriers to inclusive education for deafblind individuals. This aligns with prior research advocating for better support systems to facilitate academic integration [7]. Cognitive and educational outcomes were variably reported and often lacked pre-implant baseline data or appropriate comparison groups. As such, claims regarding cognitive improvement attributable to CI cannot be supported by the available evidence and should be interpreted as associative rather than causal.

Educational integration outcomes were modest, with only one-third of patients enrolled in mainstream schooling. This finding likely reflects systemic educational barriers and the complexity of dual sensory impairment, rather than limited auditory benefit alone. Families reported positive psychosocial outcomes in 67% of cases, consistent with studies demonstrating CI’s role in improving social participation and emotional well-being [6]. However, advanced skills such as musical perception (19%) and media engagement (14%) remained limited, suggesting that while CI restores basic auditory function, specialized training may be required to achieve higher-level auditory processing [49, 50].

High heterogeneity in outcomes like SIR (I² = 90.72%) and CAP (I² = 91.53%) reflects variability in study designs, patient demographics, and rehabilitation protocols. Funnel plot asymmetry for verbal communication and telephone competence suggests potential publication bias, possibly overestimating success rates. The predominance of observational studies and inconsistent follow-up durations (0.5–20 years) further limit the generalizability of findings. Future research should prioritize standardized outcome measures and longitudinal designs to assess long-term efficacy.

Our findings support expanding CI candidacy to include deafblind individuals, particularly those with Usher syndrome, given their comparable outcomes to non-syndromic cases. However, success depends on early implantation and multidisciplinary rehabilitation addressing both auditory and visual deficits. Future studies should investigate the impact of visual impairment severity on CI outcomes and explore innovative rehabilitation strategies, such as tactile or vibrotactile feedback, to enhance complex auditory tasks like telephone use [51].

### Strengths and limitations

This meta-analysis represents the most extensive and first evidence evaluating and filling a gap in CI in deafblind patients, gathering 36 studies and over 490 patients. Key strengths of this study encompass the large, pooled sample size, detailed outcomes stratification (speech, audiological, cognitive, educational, and psychosocial domains), providing a holistic picture of CI benefits. Subgroup analysis (Usher vs. non-Usher) and design-based comparisons that help explore effect modification and contextualize results.

However, considerable limitations should be acknowledged. First, the analysis was mainly dependent on retrospective observational studies (case series, retrospective cohorts), potentially introducing selection bias and restricting the ability to infer causality. Additionally, significant heterogeneity was identified across many outcomes, which may be attributed to methodological variability, patient characteristics, rehabilitation protocols, and differences in outcome measurement tools across studies.

### Implications for clinical practice and future directions

The evidence presented in this meta-analysis shows that cochlear implantation is an effective strategy for deafblind individuals, including those with syndromic forms like Usher syndrome, and is found to be as effective as in non-syndromic cases. It seems that earlier implantation is especially helpful as it may improve speech clarity, verbal engagement, and cognitive skills without visual stimuli. Nonetheless, enduring difficulties in verbal communication over the phone and integrated education in deafblind schooling underscore the necessity for personalized, multisensory rehabilitation approaches. Further studies are needed with clearly defined prospective designs, standardized outcome measures, extended follow-up assessments, and in-depth evaluation of visual impairment severity on functioning.

## Conclusion

This meta-analysis confirms that CI significantly improves auditory, communicative, and cognitive outcomes in deafblind individuals, though challenges remain in telephone competence and educational integration. The findings advocate for inclusive CI candidacy criteria and underscore the need for holistic, individualized rehabilitation approaches. Further research is warranted to refine patient selection, optimize post-implantation support, and develop adaptive technologies tailored to the unique needs of deafblind individuals.

## Data Availability

All data are available and attached.
